# P2X7 receptor and neuroinflammation in neurodegenerative disorders: an autoradiography study with [^18^F]JNJ-64413739

**DOI:** 10.1097/MNM.0000000000002153

**Published:** 2026-06-02

**Authors:** Abhishekh Hulegar Ashok, Sophie Field, Nisha Kuzhuppilly Ramakrishnan, Stephen Thompson, John T O’Brien, Franklin I Aigbirhio

**Affiliations:** a Department of Radiology, Addenbrooke’s Hospital, Cambridge University Hospitals NHS Foundation Trust; b Department of Radiology, University of Cambridge; c Department of Clinical Neurosciences, Molecular Imaging Chemistry Laboratory; d Department of Psychiatry, University of Cambridge School of Clinical Medicine, Level E4 Cambridge Biomedical Campus, Cambridge, UK

**Keywords:** Alzheimer’s disease, [^18^F]JNJ-64413739, positron emission tomography, neuroinflammation

## Abstract

**Background:**

Neuroinflammation plays a crucial role in neurodegenerative disorders such as Alzheimer’s disease. The P2X7 receptor (P2X7R), expressed on microglia, is involved in neuroinflammatory responses. Despite evidence of P2X7R upregulation in Alzheimer’s disease models, its role in human Alzheimer’s disease remains unclear. The PET radioligand [^18^F]JNJ-64413739 enables the assessment of P2X7R distribution in post-mortem Alzheimer’s disease brain tissue.

**Methods:**

Post-mortem brain tissue from Alzheimer’s disease and control subjects was obtained. [^18^F]JNJ-64413739 was synthesised and applied to tissue sections from the temporal and parietal cortex. Autoradiography was conducted with and without the P2X7R antagonist JNJ54173717.

**Results:**

[^18^F]JNJ-64413739 binding was observed across all brain regions, with effective blocking confirming specificity. No significant differences were found between Alzheimer’s disease and controls in the temporal (*P* = 0.84) or parietal cortex (*P* = 0.90) in the first experiment. The second experiment, using a modified protocol also did not reveal a significant difference between controls and Alzheimer’s disease in either temporal (*P* = 0.66) or parietal cortex (*P* = 0.38). White matter exhibited significantly higher binding than grey matter (*P* < 0.01), but no disease-specific differences were noted.

**Conclusion:**

This study demonstrates P2X7 receptor-specific binding of [^18^F]JNJ-64413739 but finds no significant differences between post-mortem tissue of Alzheimer’s disease cases and controls. These findings suggest that while the tracer shows promising in vitro characteristics, the role of P2X7R in Alzheimer’s disease pathology and its utility as a biomarker require further validation through in vivo imaging studies across disease stages.

## Introduction

Neurodegenerative disorders such as Alzheimer’s disease are, and will remain, major challenges for public health in the coming years because of the ageing population. The estimated cost of dementia to the UK is currently £42 billion a year [1]. Neuroinflammation is increasingly recognized as an important mechanism that may be key to triggering and accelerating degenerative diseases such as Alzheimer’s disease. The microglial dysfunction resulting in inadequate clearance of amyloid-beta along with increased secretion of cytokines has been hypothesised to be a key molecular mechanism underlying pathogenesis of Alzheimer’s disease [2]. Specifically, activated microglial activity is crucial early in the course of illness, such as during the prodromal or ‘mild cognitive impairment’ stage [3].

The current in vivo evidence on neuroinflammation in Alzheimer’s is largely based on translocator protein (18 kDa) (TSPO) PET imaging methods. However, not all studies using the widely used [^11^C]PK11195 radiotracer or second-generation TSPO tracers such as [^11^C]PBR28 have detected a difference between groups [4]. We have demonstrated increased binding using [^11^C]PK11195 PET brain imaging in Alzheimer’s disease and its prodrome, amyloid positive mild cognitive impairment, in mild dementia with Lewy bodies (DLB), frontotemporal dementia and Parkinson’s associated dementia [3,5–9]. However, the TSPO tracers have several methodological limitations such as low signal-to-noise ratio, endothelial binding and genotype effects [10]. Hence there has been an active effort in recent years to develop alternative PET-based approaches for imaging neuroinflammation [11,12].

One of these approaches is based on imaging the role of purinergic system, specifically the P2X7 receptor, in neuroinflammation [13–17]. Ionotropic P2X7 receptors are expressed on monocytes, macrophages, and microglia in the rat and human brain [18–20]. Activation of the P2X7 receptor is associated with release of pro-inflammatory molecules such as TNF-α and interleukin-1β, and induction of apoptosis [21–23]. The P2X7 receptor is upregulated near Aβ plaques in an APPPS1 animal model of Alzheimer’s disease [20]. Similarly, cultured foetal human microglia cells exposed to Aβ1-42 had significantly elevated levels of P2X7 receptor (by 106%) compared with untreated cells [24]. P2X7 knockout model showed greater accumulation of IL-1β following intra-hippocampal injection of Aβ compared with wild-type [25]. The P2X7 receptor mediates superoxide production in primary microglia and results in neuronal death [26]. Studies have also shown that circadian cytosolic calcium oscillations depend on activation of the P2X7 receptor, and it is disrupted in the presence of Aβ [27]. In addition, neuroinflammation induced by Aβ peptide has been reported to change the P2X7 receptor distribution pattern, specifically increasing its expression at advanced and late stages, when microgliosis occurs, but not in the early stages [28].

Several animal studies have demonstrated that blocking P2X7 receptor can reduce amyloid deposition. In a transgenic mouse model of familial Alzheimer’s disease expressing human amyloid precursor protein mutant protein, inhibition of the P2X7 receptor significantly decreased the number and size of hippocampal amyloid plaques [29]. A 2-hour priming of the microglial cells with Apo-serum amyloid A, followed by addition of ATP resulted in release of IL-1β and a selective P2X7 receptor antagonist blocked release of IL-1β [30].

Several tracers have been developed to assess P2X7 receptors in vivo. A study using [^11^C]JNJ-717 tracer in amyotrophic lateral sclerosis found no statistically significant difference between patients and controls [31]. [^18^F]GSK1482160 PET revealed elevated cerebral P2X7 receptor in a tauopathy model [32].

A recently developed P2X7 tracer is [^18^F]JNJ-64413739 for which preclinical studies have shown an affinity of 15.9 nM for the human P2X7 receptor and excellent target engagement [33,34]. In models of epilepsy, [^18^F]JNJ-64413739 uptake was shown to correlate with severity of status epilepticus and peripheral inflammation [35]. Further, P2X7 receptor antagonism was found to reduce post-traumatic hyperexcitability in a murine traumatic brain injury model and reduced activation of P2X7 receptor as measured by [^18^F]JNJ-64413739 [36], suggesting therapeutic potential. A recent autoradiography of human brain tissue confirmed specific regional binding [37]. Test–retest PET studies in healthy human subjects determined it has a variability of 10.7% (SD 2.2%) based on a two-tissue compartment model *V*_T_ [38].

Despite convincing evidence in preclinical models, in vitro studies have not investigated P2X7 levels in Alzheimer’s disease. In this study, we evaluated the feasibility of assessing P2X7 receptor binding in vitro in the post-mortem brain tissue from individuals with Alzheimer’s disease using [^18^F]JNJ-64413739.

## Methods

### Tissue

This study was conducted in accordance with the principles of the Declaration of Helsinki. Human brain tissue was obtained from the Newcastle Brain Tissue Resource (Newcastle Brain Bank). Ethical approval for the use of post-mortem human tissue was granted under the Newcastle Brain Bank ethical approval and committee review by the North East – Newcastle & North Tyneside 1 Research Ethics Committee (REC reference: 19/NE/0008, IRAS ID: 255808). All donors had provided informed consent for the use of their tissue in research, in accordance with the policies of the brain bank.

Sectioning was carried out at Cambridge Brain Bank, producing approximately 10 μm thick slices taken from posterior temporal, inferior parietal cortex and caudate where NIMROD study [3] has shown significantly higher inflammation in subjects with Alzheimer’s disease than healthy control. The groups included subjects with Alzheimer’s disease, DLB, and healthy controls.

### Radiochemical synthesis of [^18^F]JNJ-64413739

**Fig. 1:**
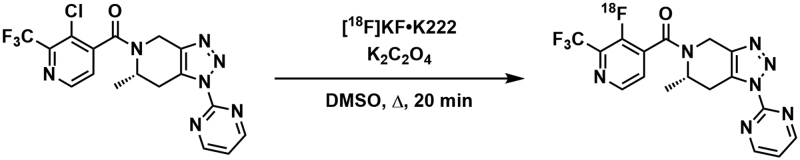
Radiochemical synthesis of [^18^F]JNJ-64413739 from the corresponding 3-chloropyridinyl precursor by halogen exchange.

[^18^F]JNJ-64413739 was synthesised using a GE FX_FN_ module (GE Healthcare, Chicago, IL, USA) (Experiment 1) or a Synthra RNplus module (Synthra, Hamburg, Germany) (Experiment 2) from the corresponding 3-chloropyridinyl precursor (JNJ-64410047, ENIGMA Biomedical, Knoxville, Tennessee, USA)), by a halogen exchange reaction with [^18^F]fluoride. Briefly, [^18^F]fluoride was azeotropically dried in the presence of potassium oxalate and Kryptofix-222 (Merck, Cambridge, UK ), before being heated with the 3-chloropyridinyl precursor in anhydrous DMSO (Sigma-Aldrich, Gillingham, UK) (Fig. 1), using a modified procedure to that reported previously [33]. After cooling and dilution, the radiolabelled product was isolated by semi-preparative HPLC, and formulated by solid-phase extraction into 10% v/v ethanol in saline solution. For experiment 1, [^18^F]JNJ-64413739 was isolated in 3.3 ± 1.1% decay corrected radiochemical yield (0.24–0.61 GBq isolated) with more than 99% radiochemical purity in approximately 80 min from delivery of the [^18^F]fluoride to the module. The average molar activity obtained for the isolated [^18^F]JNJ-64413739 was 7.0 ± 2.3 GBq/µmol (*n* = 4) at end-of-synthesis. For experiment 2, [^18^F]JNJ-64413739 was isolated in 2.8 ± 0.3% decay corrected radiochemical yield (0.41–0.88 GBq isolated) with more than 99% radiochemical purity in approximately 100 min from delivery of the [^18^F]fluoride to the module. The average molar activity obtained for the isolated [^18^F]JNJ-64413739 was 9.9 ± 5.7 GBq/µmol (*n* = 2) at end-of-synthesis. The final radiolabelled product was diluted as appropriate for autoradiography studies.

### Autoradiography

Experiment 1 autoradiography: autoradiography was performed in accordance with previously established method by Ory *et al*. [34]. The brain slices were washed in Tris-HCl 50 mM buffer (pH 7.4) for 10 min (2×) at room temperature and dried. Then brain sections were incubated with [^18^F]JNJ-64413739, 0.1–0.2 MBq/ml in Tris buffer pH 7.4 or tracer in the presence of 10 μM of the P2X7R antagonist reference compound JNJ-64413739 for 60 min. After the incubation, the brain sections were washed twice for 5 min in ice-cold Tris-HCl 50 mM buffer (pH 7.4). After a dip in purified ice-cold water, the slides were dried. Autoradiograms were obtained by exposing the slides overnight to a high-performance phosphor storage screen. The screens were read using GE CR-reader. The data were quantified using advanced image data analyzer version 5.1. The radioactivity concentration in the autoradiograms were expressed as intensity-background [QL]/area [pixel].

Experiment 2a autoradiography: the protocol was modified from an established protocol used in the MICL lab. Prior to using human brain tissue, several optimisation experiments using rat brain tissue were conducted. A more recent study [37] used an alternative incubation buffer with addition of EGTA and MgCl_2_. EGTA in the buffer mimics a living cell environment but chilates magnesium ions; therefore, MgCl_2_ is added. We tested both Tris-HCL and EGTA-based buffers and found no improvement in the image quality or background levels. Thus we continued using Tris-HCl as a more convenient bufffer. The RADPro calculator was used to predict future activity levels, allowing precise determination of the time point at which the tracer would decay to the target activity. An activity of 0.5 MBq was determined to be optimal and was therefore used for the human tissue experiments.

### Experiment 2b human tissue protocol

The brain slides were left to defrost for 20 min before washing in Tris-HCl 50 mM + 0.5% BSA buffer (pH 7.4) for 10 min (2×) at room temperature. The slides were incubated for 60 min at 37 °C with either 1 ml [^18^F]JNJ-64413739 in Tris-HCl 50 mM + 0.5% BSA at 0.5 MBq/ml or the tracer with 10 μM of the P2X7R antagonist reference compound JNJ-64413739 for blocking. After the incubation, the brain slides were washed for 5 min (twice) in ice-cold Tris-HCl 50 mM buffer (pH 7.4). Slides were then dipped briefly in ice-cold deionized water before being dried. Slides were exposed overnight to a high-performance phosphor storage screen and read the next day using GE CR-reader. Advanced image data analyzer version 5.1 was used to quantify the data of the autoradiograms and prepare it for statistical analysis. A serial dilution series was created using a thin layer chromatography plate in order to create a calibration curve to estimate the number of Becquerels (Bq – the unit for radioactivity). The radioactivity values were expressed as intensity-background [QL]/area [pixel] due to technical failure of the calibration curves.

### Genotyping

Previous studies have reported a possible genotype effect of P2X7 receptor binding [39]. To assess this confounder, rs3751143 genotyping was performed using Kompetitive allele-specific PCR (KASP) genotyping technology developed at LGC (https://www.lgcgroup.com). The KASP technology is based on allele-specific PCR, which enables the bi-allelic scoring of single nucleotide polymorphisms, insertions and deletions. The single nucleotide polymorphism-specific KASP assay and the universal KASP-TF assay mix are added to the DNA in 1536-well plates and a series of 35 cycles of PCR performed. On completion of the 35 cycles the plates were read using a fluorescence plate reader and visually inspected to assess the progression of the PCR reaction, further cycles were performed if it was decided that the PCR reaction has not reached its endpoint. Once the reaction has reached its endpoint, the data were examined, and genotypes assigned.

### Statistical analysis

Statistical analysis was performed with SPSS (IBM, version 29; IBM Corp., Armonk, New York, USA) for MAC OS X and GraphPad Prism version 9 (GraphPad Software, Boston, Massachusetts, USA). Normality of distribution was tested using the Shapiro–Wilk test. The main hypothesis that there was a group difference in the P2X7 availability in the temporal and parietal cortex, was tested using an independent-sample t-test. For experiment 2, statistical analysis was performed using ANOVA package in RStudio. All data are presented as mean ± SD. The significance level α was set for all comparisons at *P* < 0.05.

## Results

In first experiment, five patients with Alzheimer’s disease, five controls, and one patient with DLB was included. There was a technical failure in the experiment of temporal cortex experiment of two patients with Alzheimer’s disease. Final data were available for the following groups: temporal cortex – three patients with Alzheimer’s disease, one DLB, and five controls; parietal cortex – five patients with Alzheimer’s disease, one DLB, and five controls; Caudate – one patient with DLB and one control. In the second experiment, eight patients with Alzheimer’s disease and eight controls were included, with partial overlap of samples from the first experiment.

Demographic details are available in Table 1.

**Table 1:** Demographic details of subjects included

Parameter	AD (*n *= 8)	Controls (*n* = 8)	*P*-value
Age (mean ± SD)	78.2 ± 7.7	89.6 ± 7.6	0.009
Gender (F:M)	4 : 4	5 : 3	0.61
Post-mortem delay in hours (mean ± SD)	23.6 ± 10.3	16.9 ± 5.1	0.12
Tau Braak staging 1 2 6	0 0 8	2 6 0	<0.001
MMSE close to death	11.7 ± 9.5	25.8 ± 4	0.002
rs3751143 genotyping A:A A:C C:C	6 2 0	5 2 1	0.714

AD, Alzheimer’s disease.

**Fig. 2:**
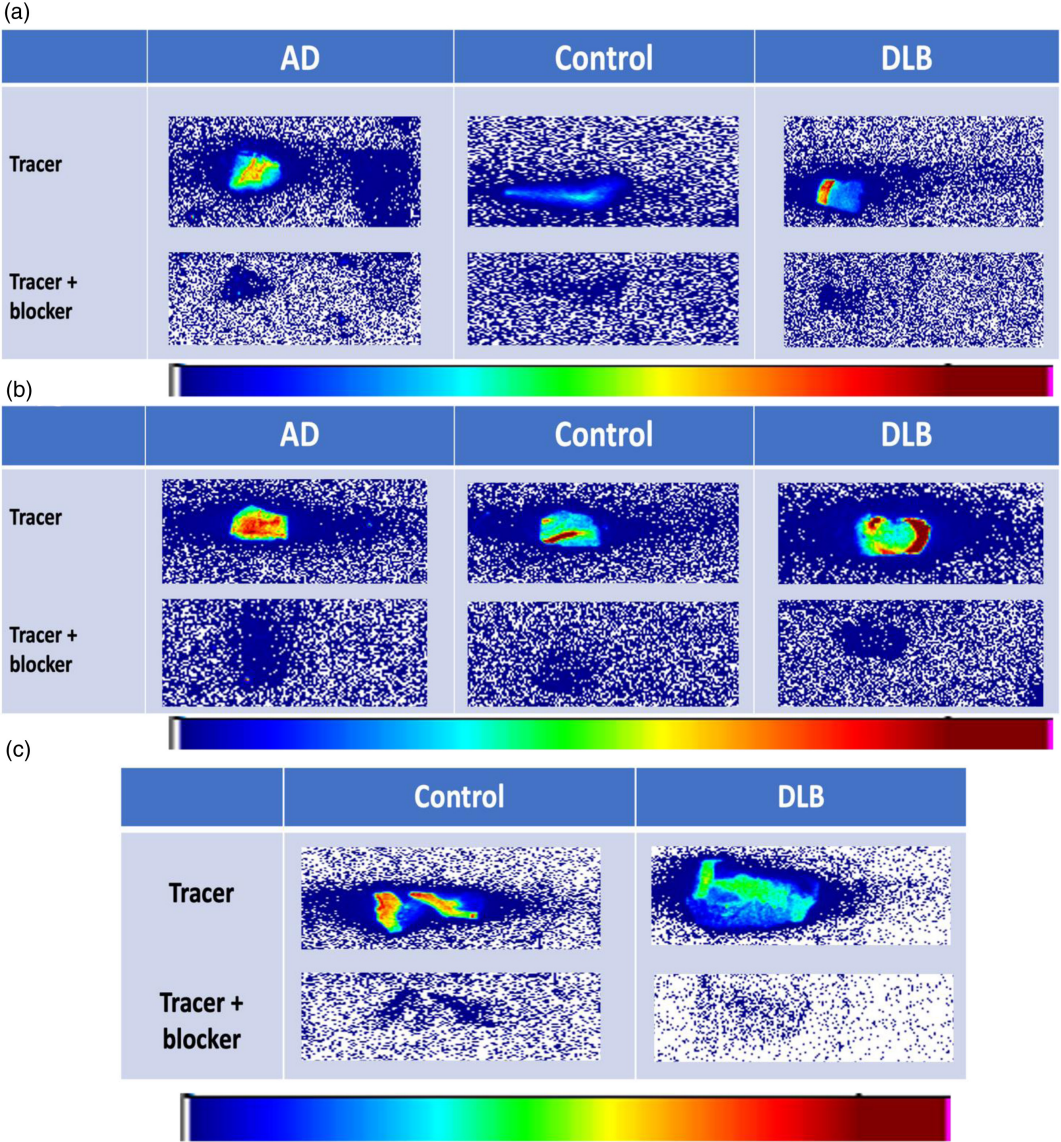
Representative autoradiogram of (a) parietal cortex; (b) temporal cortex; and (c) caudate showing blocking in all groups. AD, Alzheimer’s disease, DLB, Lewy body dementia.

**Fig. 3:**
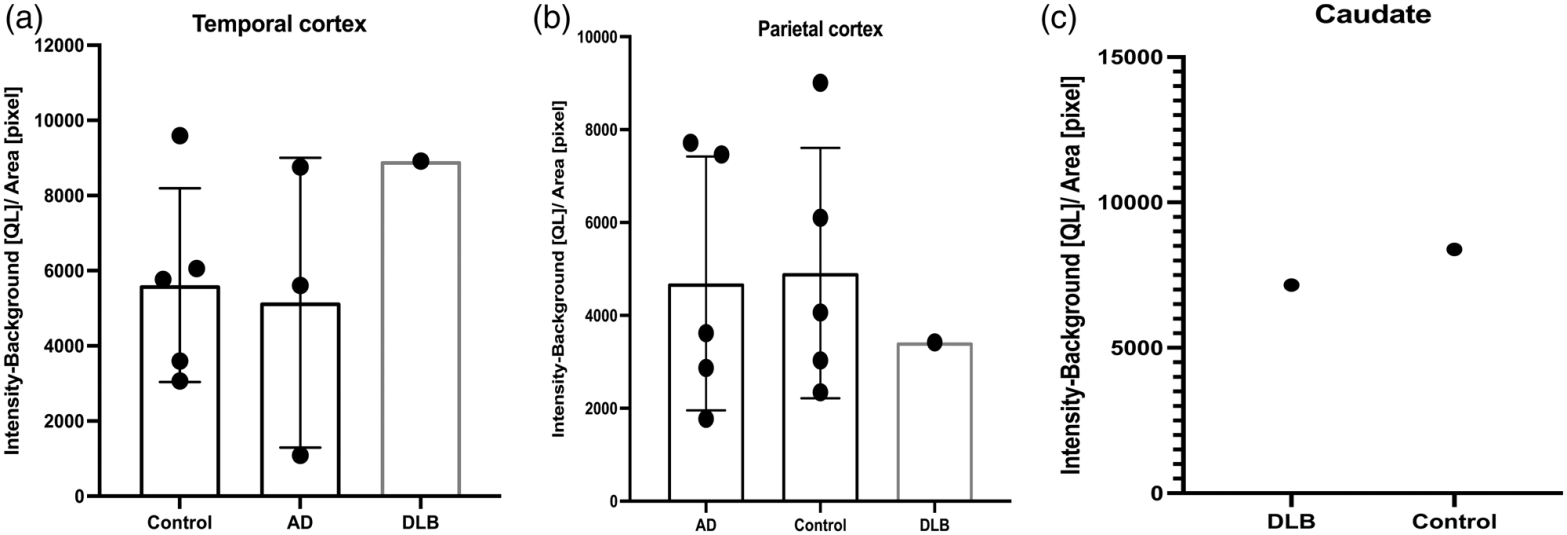
Findings from experiment 1 showing [^18^F]JNJ-64413739 binding in patients with AD and healthy controls. (a) Temporal cortex and (b) parietal cortex [^18^F]JNJ-64413739 binding in patients with AD and healthy control. No statistically significant difference was noted. AD, Alzheimer’s disease, control, healthy control.

In experiment 1, five patients with Alzheimer’s disease and five healthy controls were age-matched (mean age ± SD: AD = 83 ± 5.3 years; controls = 85.8 ± 6.3 years; *P* = 0.47). In experiment 2, age matching was not feasible due to limitations in post-mortem tissue availability. Representative autoradiogram of temporal and parietal cortex showing blocking in all cohorts is shown in Fig. 2. In our autoradiography experiments, tissue sections were incubated with the radiotracer in the presence of the P2X7R antagonist JNJ-64413739. The presence of the antagonist markedly reduced tracer binding, demonstrating that the signal was largely displaceable and therefore specific to the P2X7 receptor. Representative autoradiograms showing tracer binding and blockade by the antagonist are presented in Fig. 2, illustrating minimal non-specific binding. Figure 2c shows autoradiogram of caudate in patient with DLB. There was no statistically significant difference in the temporal cortex binding between Alzheimer’s disease and controls (*t* = 0.2, df = 6, *P* = 0.84, Fig. 3a). Similarly, there was no statistically significant difference in the parietal cortex binding between Alzheimer’s disease and controls (*t* = 0.1, df = 8, *P* = 0.9, Fig. 3b).

**Fig. 4:**
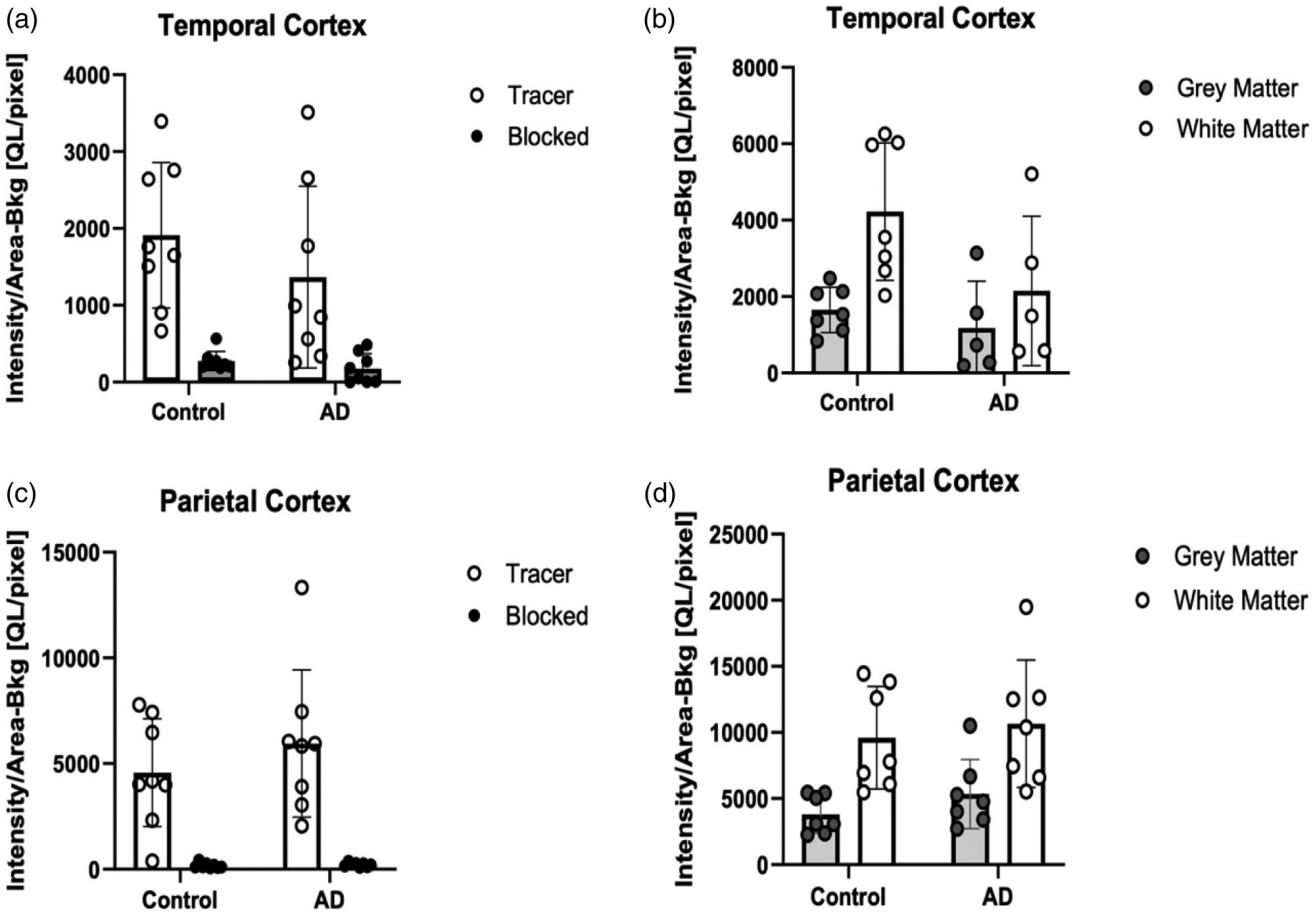
Findings from experiment 2 showing [^18^F]JNJ-64413739binding in patients with AD and healthy controls. (a) Temporal cortex binding and blocking property; (b) temporal cortex binding in grey and white matter; (c) parietal cortex binding and blocking property; (d) parietal cortex binding in grey and white matter. No statistically significant difference was noted. AD, Alzheimer’s disease, control, healthy controls.

**Fig. 5:**
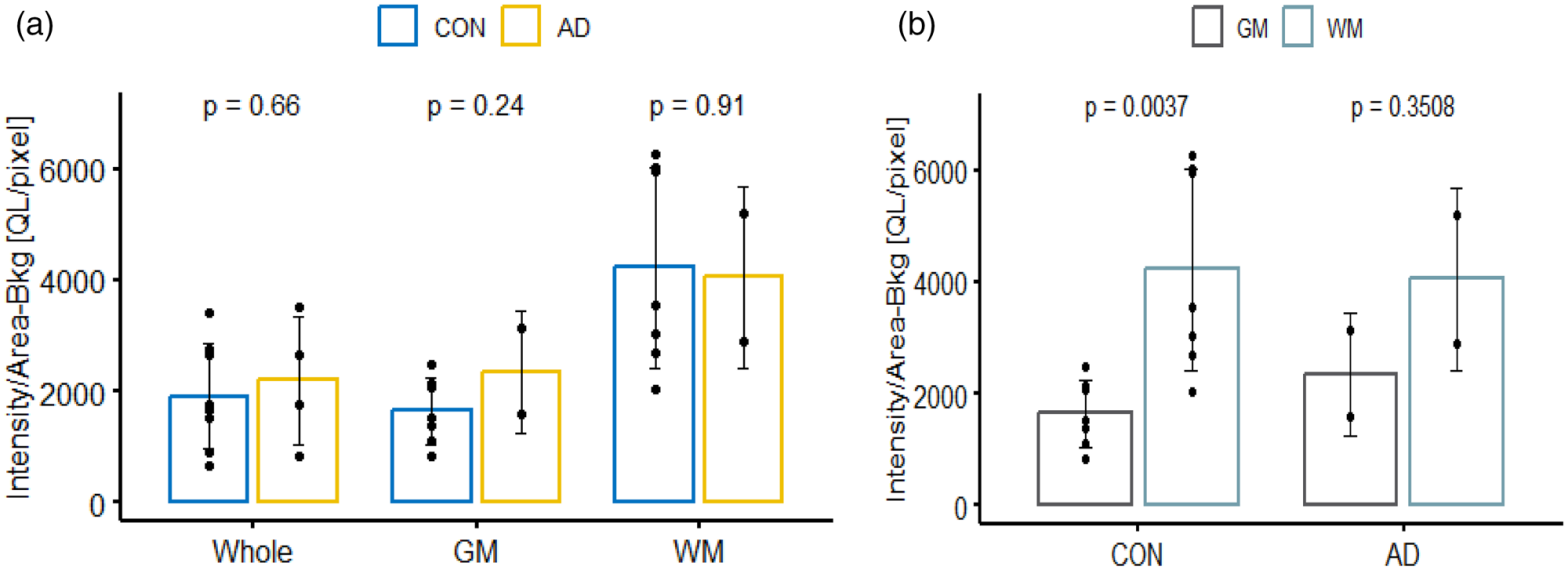
Temporal cortex [^18^F]JNJ-64413739 binding to P2X7 in patients with AD and healthy controls. (a) Comparisons of intensity-background [QL]/area [pixel] between healthy controls and AD (whole: con *n* = 8, AD *n* = 8, GM/WM = con = 7, AD *n* = 2); (b) comparisons of intensity-background [QL]/area [pixel] between grey and white matter within each disease group, (GM/WM = con = 7, AD *n* = 2); results were analysed using ANOVA package in R. AD grey and white matter were excluded from statistical analysis due to small sample size (*n* = 2). AD, Alzheimer’s disease; CON, control; GM, grey matter; WM, white matter.

**Fig. 6:**
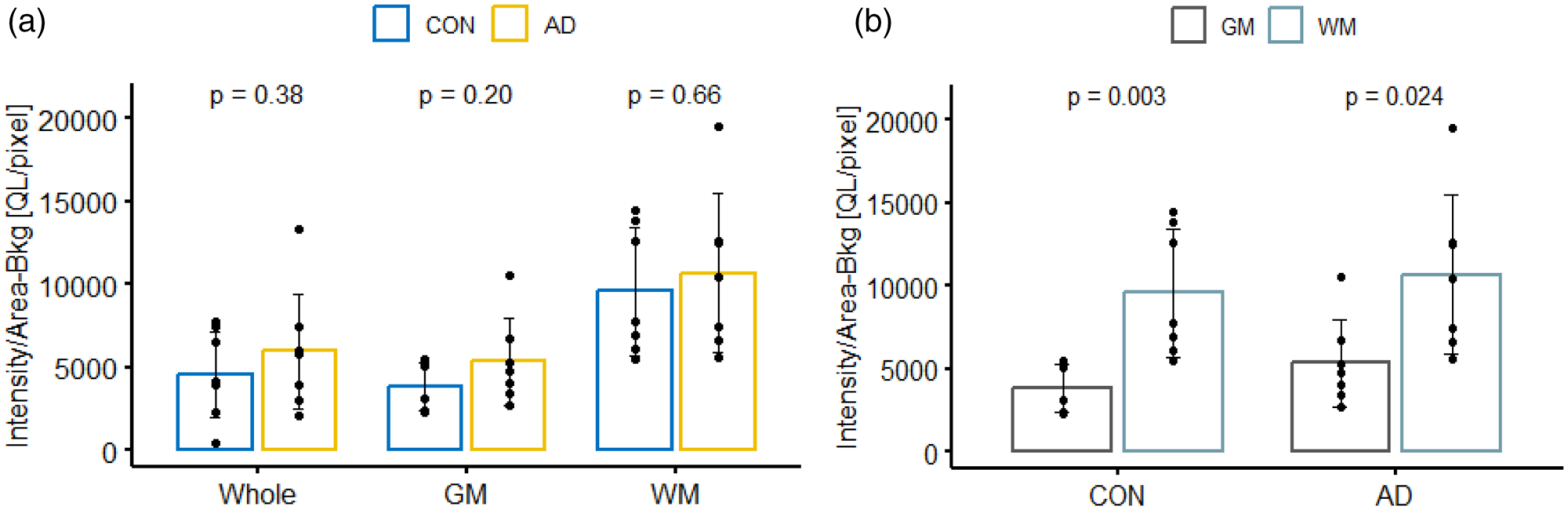
Inferior parietal cortex [^18^F]JNJ-64413739 binding to P2X7 in patients with AD and healthy controls. (a) Comparisons of intensity-background [QL]/area [pixel] between healthy controls and AD (whole: con *n* = 8, AD *n* = 8, GM/WM = con = 7, AD *n* = 7); (b) comparisons of intensity-background [QL]/area [pixel] between grey and white matter within each disease group (GM/WM = con = 7, AD *n* = 7), results were analysed using ANOVA package in R. AD, Alzheimer’s disease; CON, control; GM, grey matter; WM, white matter.

No significant differences were observed between controls and Alzheimer’s disease in the analysis in either temporal (*P* = 0.66) or parietal cortex (*P* = 0.38) (Fig. 5). Significant differences were not observed between disease groups in either grey or white matter regions or the whole region (Fig. 4). Significant differences were apparent between grey and white matter within disease groups (Fig. 5b and Fig. 6b); controls had significant increased signal in temporal (*P* = 0.004) and inferior parietal (*P* = 0.003) white matter brain regions compared to grey matter and similar findings were seen in Alzheimer’s disease in the inferior parietal (*P* = 0.024).

## Discussion

To our knowledge, this is the first study investigating P2X7 availability in brain tissue from patients with Alzheimer’s disease, DLB, and healthy controls using [^18^F]JNJ-64413739. Our study shows that [^18^F]JNJ-64413739 was consistently blocked across the experimental groups suggesting low non-specific binding. However, we could not find a statistically significant difference between the groups. Our results are consistent with another study using [^11^C]SMW139 [40], which did not demonstrate significant differences in P2X7 level as measured by the PET tracer between patients with Alzheimer’s disease and controls. This contrasts with evidence from mRNA and protein level studies where there was significant upregulation of P2X7 levels in patients compared to controls [20,41].

Over the past two decades, PET imaging studies of neuroinflammation have predominantly focussed on the TSPO as the principal molecular target. As a result, there has been increasing interest in exploring alternative molecular targets beyond TSPO for PET imaging of neuroinflammation. TSPO PET imaging, despite known limitations such as genotype sensitivity and off-target binding, has outperformed P2X7 PET imaging in detecting neuroinflammation in certain neurodegenerative conditions. For example, in vivo PET studies demonstrated approximately 13% higher [^18^F]DPA-714 binding in the motor cortex of patients with amyotrophic lateral sclerosis, whereas [^11^C]JNJ-717 PET failed to significantly differentiate amyotrophic lateral sclerosis patients from healthy controls [31]. If a similar lack of difference is observed between Alzheimer’s disease patients and controls in human studies, one plausible explanation is that P2X7 PET may be inherently less sensitive to chronic or low-grade neuroinflammation than TSPO PET. TSPO serves as a more generalised marker of glial activation, expressed by both microglia and astrocytes, whereas P2X7 receptor expression is more restricted and may only be markedly upregulated during specific inflammatory states involving sustained ATP signalling and inflammasome activation. Accordingly, the absence of a detectable P2X7 signal difference should not necessarily be interpreted as absence of neuroinflammation but rather may indicate that the inflammatory state present is not one in which P2X7 is sufficiently upregulated to provide measurable contrast. Consequently, in conditions where microglial activation occurs without strong engagement of the P2X7 pathway, TSPO tracers may detect increased binding while P2X7-targeted tracers remain at baseline levels.

This pattern of divergence between preclinical expectations and in vivo PET results is not unique to P2X7. For instance, the cannabinoid type 2 receptor tracer [^11^C]NE40, despite promising preclinical and post-mortem data, also failed to show increased binding in Alzheimer’s disease patients. Instead, [^11^C]NE40 PET revealed lower cannabinoid type 2 receptor availability in vivo in Alzheimer’s disease, with no clear relationship to Aβ plaque burden [42]. Taken together, these findings suggest that not all neuroimmune targets demonstrate robust or directionally consistent changes on PET imaging in dementia, underscoring the complexity of translating molecular neuroinflammatory markers into reliable in vivo biomarkers [43].

Previous studies have shown that P2X7 receptors are more widely expressed in white matter than grey matter [38]. Our analysis did not detect a statistical difference in the distribution of P2X7 in the grey and white matter between groups. Some studies have shown that P2X7 levels quantified by PET may be influenced by genotype [39], however, our genotype distribution did not differ significantly between Alzheimer’s disease and control groups. Nevertheless, our sample size was limited, and further studies with larger cohorts are needed to clarify the potential impact of genetic variants.

Our study has some limitations. Firstly, preclinical evidence shows that P2X7 is expressed in areas with high beta-amyloid deposition [28] and we have not quantified beta-amyloid load in the area where autoradiography was performed. It is possible that the tissue we studied might have had reduced beta-amyloid load. Second, there was a statistically significant difference in age between patients with Alzheimer’s disease and controls. This was due to availability of the brain tissue from the brain bank. Our results remained valid after statistically controlling for the effects of age. Future studies should evaluate these factors more rigorously.

Immunohistochemical validation was not performed in this study, which represents a limitation. Direct assessment of P2X7 receptor expression using immunohistochemistry (e.g. P2X7R-specific antibodies or microglial markers such as Iba-1) could have provided complementary information to support the autoradiography findings and allowed spatial correlation between receptor expression and radiotracer binding. The absence of such histopathological confirmation limits our ability to determine whether subtle differences in P2X7R expression may exist between disease and control tissues that were not detectable by autoradiography alone. Future studies should incorporate immunohistochemistry or similar histological approaches to verify receptor density and better characterize the relationship between P2X7R expression and tracer binding in neurodegenerative disease.

Our study showed that [^18^F]JNJ-64413739 had excellent specificity; however, the absence of detectable disease-related differences highlights the importance of disease stage, regional pathology, and complementary imaging targets to evaluate neuroinflammation. This study underscores the feasibility of using [^18^F]JNJ-64413739 autoradiography in human brain tissue and informs future in vivo imaging studies aiming to characterize P2X7 receptor dynamics in neurodegenerative disease.

## Acknowledgements

This work was funded by Alzheimer’s Research UK East Network Pump Priming Grant to A.H.A. This research was also supported by the NIHR Cambridge Biomedical Research Centre to F.I.A. The views expressed are those of the author(s) and not necessarily those of the NIHR or the Department of Health and Social Care. Material for the radiosynthesis of [^18^F]JNJ-64413739 and chemical standards was supplied by ENIGMA Biomedical group, Inc.

A.H.A. conceptualized the study, led the study design, collected data, performed analysis and wrote first draft. S.F. and N.K.R. contributed to data acquisition and analysis. S.T. provided technical expertise in radiosynthesis and performed experiments. J.T.O. and F.I.A. provided senior supervision, critical input on study design, and contributed to manuscript revision. All authors reviewed and approved the final manuscript.

All participants provided written consent.

The data supporting the findings of this study are available from the corresponding author upon reasonable request. Use of human tissue was governed by the terms of access set by the Newcastle Brain Bank.

### Conflicts of interest

J.T.O. has acted as a consultant for TauRx, Novo Nordisk, Biogen, Roche, Lilly, GE Healthcare, and Okwin and received grants or academic in kind support from Avid/Lilly, Merck, UCB, and Alliance Medical. He is supported by the NIHR Cambridge Biomedical Research Centre and the Medical Research Council funded Dementias Platform UK. F.I.A. supported by the NIHR Cambridge Biomedical Research Centre and the Medical Research Council funded Dementias Platform UK. None of the authors have any relevant disclosures. For the remaining authors, there are no conflicts of interest.
